# Investigating the effect of Area Deprivation Index on the association of *APOE‐*ε4 and *APOE*‐ε2 with Alzheimer’s disease in a Multiethnic Autopsy Cohort

**DOI:** 10.1002/alz.092762

**Published:** 2025-01-03

**Authors:** Geovanna Yepez‐Sisalema, Lindsey A Kuchenbecker, Aishwarya Pradeep, Rous M Gonzalez‐Soto, Thuy Nguyen, Katie D Sotelo, Michael G. Heckman, Joseph S. Reddy, Mariet Allen, Geidy E Serrano, Thomas G. Beach, Stefan Prokop, Dennis W. Dickson, Nilüfer Ertekin‐Taner, Minerva M. Carrasquillo

**Affiliations:** ^1^ Mayo Clinic, Jacksonville, FL USA; ^2^ Banner Sun Health Research Institute, Sun City, AZ USA; ^3^ University of Florida, Gainesville, FL USA

## Abstract

**Background:**

African Americans (AA) and Latin Americans (LA) are at a higher risk of developing AD compared to non‐Hispanic whites (NHW) but are traditionally underrepresented in AD research. The disproportionate risk is likely multifactorial including differences in co‐morbidities and structural and social determinants of health (SSDoH). AD risk is thought to result from multiple genetic and environmental factors, and their interactions (GxE). However, the molecular mechanisms underlying these interactions are largely unknown. The best‐established genetic risk factor for AD is *APOE*‐ε4, while *APOE*‐ε2 is protective. Environmental factors, such as living in neighborhoods with greater socioeconomic disadvantage, have been associated with greater AD neuropathology and worse cognitive function. Studying the potential modifying role of neighborhood disadvantage regarding associations of *APOE*‐ε4 and *APOE*‐ε2 with AD risk using a socioeconomic metric like the Area Deprivation Index (ADI) in a multiethnic post‐mortem cohort could offer valuable insights for precision medicine.

**Method:**

We analyzed a post‐mortem cohort of 421 multi‐ethnic donors (NHW: N = 234, AD = 70, non‐AD = 164; LA: N = 145, AD = 102, non‐AD = 43; AA: N = 42, AD = 24, non‐AD = 18), from Mayo Clinic Florida, Banner Sun Health Research Institute, and the University of Florida Brain Banks as part of the AMP AD Diversity initiative. This study used the same neuropathological criteria to categorize AD vs. non‐AD as in the MayoRNAseq study (syn5550404). ADI from the Neighborhood Atlas® was obtained from de‐identified addresses. Associations of *APOE*‐ε4, *APOE*‐ε2, and ADI with AD risk were examined using logistic regression models adjusted for age‐at‐death, sex, and ethnic group; interactions of *APOE* genotype with ADI were also assessed.

**Result:**

*APOE*‐ε4 and *APOE*‐ε2 showed a significant association with AD diagnosis (*APOE*‐ε4_combined cohort_ OR = 3.23, P = 3.14E‐08; *APOE*‐ε2_combined cohort_ OR = 0.30, P = 0.003). However, contrary to prior findings, our study found no association between ADI and AD diagnosis [OR = 1.023 (per each 10 unit increase in ADI), P = 0.629]. No significant interactions of ADI with *APOE*‐ε4 or *APOE*‐ε2 regarding association with AD diagnosis were identified (P = 0.083, P = 0.781).

**Conclusion:**

This is the first study evaluating the potential interaction of neighborhood disadvantage with *APOE* on AD risk in a multi‐ethnic autopsy cohort. However, these findings require further investigation in larger, more well‐powered cohorts for validation.

